# MiR-1268b confers chemosensitivity in breast cancer by targeting ERBB2-mediated PI3K-AKT pathway

**DOI:** 10.18632/oncotarget.20099

**Published:** 2017-08-09

**Authors:** Wen-Jie Zhu, Xu Chen, Ya-Wen Wang, Hai-Ting Liu, Ran-Ran Ma, Peng Gao

**Affiliations:** ^1^ Department of Pathology, Qilu Hospital, Shandong University, Jinan, P.R. China; ^2^ Department of Pathology, School of Medicine, Shandong University, Jinan, P.R. China

**Keywords:** MiR-1268b, breast cancer, chemoresistance, ERBB2, PI3K-AKT

## Abstract

Chemoresistance represents a major obstacle to effective therapy for breast cancer. Emerging evidences associated aberrantly expressed miRNAs with tumor development and chemoresistance. MiR-1268b has never been studied in any cancers before, and its roles in mediating tumor progression and drug resistance are still unclear. Selected from miRNA microarray and confirmed by real-time quantitative PCR (RT-qPCR), miR-1268b was found to be significantly upregulated in drug sensitive and ERBB2 negative tissues, as well as in breast cancer patients with low clinical stage. And miR-1268b had a higher expression in chemosensitive breast cancer cell lines, compared with the chemoresistant cell line. Moreover, the results revealed that miR-1268b induced breast cancer cell apoptosis and increased cell chemosensitivity. ERBB2 was demonstrated to be the target gene of miR-1268b by dual-luciferase reporter assays, western blot, and immunocytochemistry. Furthermore, PI3KCA, AKT, BCL2 in the ERBB2-PI3K-AKT signaling pathway were found to be downstream effectors of miR-1268b. In conclusion, miR-1268b increased chemosensitivity, at least in part, via modulation of PI3K-AKT pathway by targeting ERBB2. MiR-1268b may serve as a potential therapeutic target for patients with breast cancers.

## INTRODUCTION

According to the data from the American Cancer Society, breast cancer remains the most common malignant tumor, accounting for 29% of all new carcinoma cases and the leading lethal cancer-related mortality in female worldwide [1]. In addition to surgical resection and ionizing radiation, chemotherapy is an efficiency treatment for patients with breast cancers [2]. However, there are a lot of patients who experience treatment failure due to chemoresistance [3]. Therefore, it is essential to discover informative gene biomarkers for predicting effective therapy targets to conquer drug resistance [4].

In recent years, more and more non-coding RNAs, especially microRNAs, are found to be associated with the development and treatment of cancers. MiRNAs are a species of endogenous short non-coding RNAs that participate in many aspects of homeostasis and cellular differentiation, acting as mediators of gene expression by binding to their 3’-untranslated regions (UTRs) [5]. Using a miRNA microarray, we revealed that miR-1268b was significantly upregulated in chemosensitive tissues from patients with breast cancer compared with the chemoresistant ones, indicating that miR-1268b may be associated with the chemosensitivity of breast cancer.

ERBB2 (HER-2) belongs to the HER family of receptor tyrosine kinases [6], which is commonly overexpressed and amplified in various tumors. In breast cancer, it was reported that nearly 30% cases showed ERBB2 overexpression. Notably, ERBB2 overexpression in breast cancer has become an independent diadynamic criterion of worse prognosis. And many researches demonstrated that ERBB2 was associated with chemoresistance of breast cancer [7–9]. At present, the underlying mechanisms of ERBB2 rendering cancer cells chemoresistance remain largely unclear. In this research, we demonstrate, for the first time, that miR-1268b is involved in the regulation of breast cancer chemosensitivity and apoptosis by targeting ERBB2.

## RESULTS

### The expression of miR-1268b in human breast cancer tissues and its association with clinicopathologic parameters

**Figure 1:**
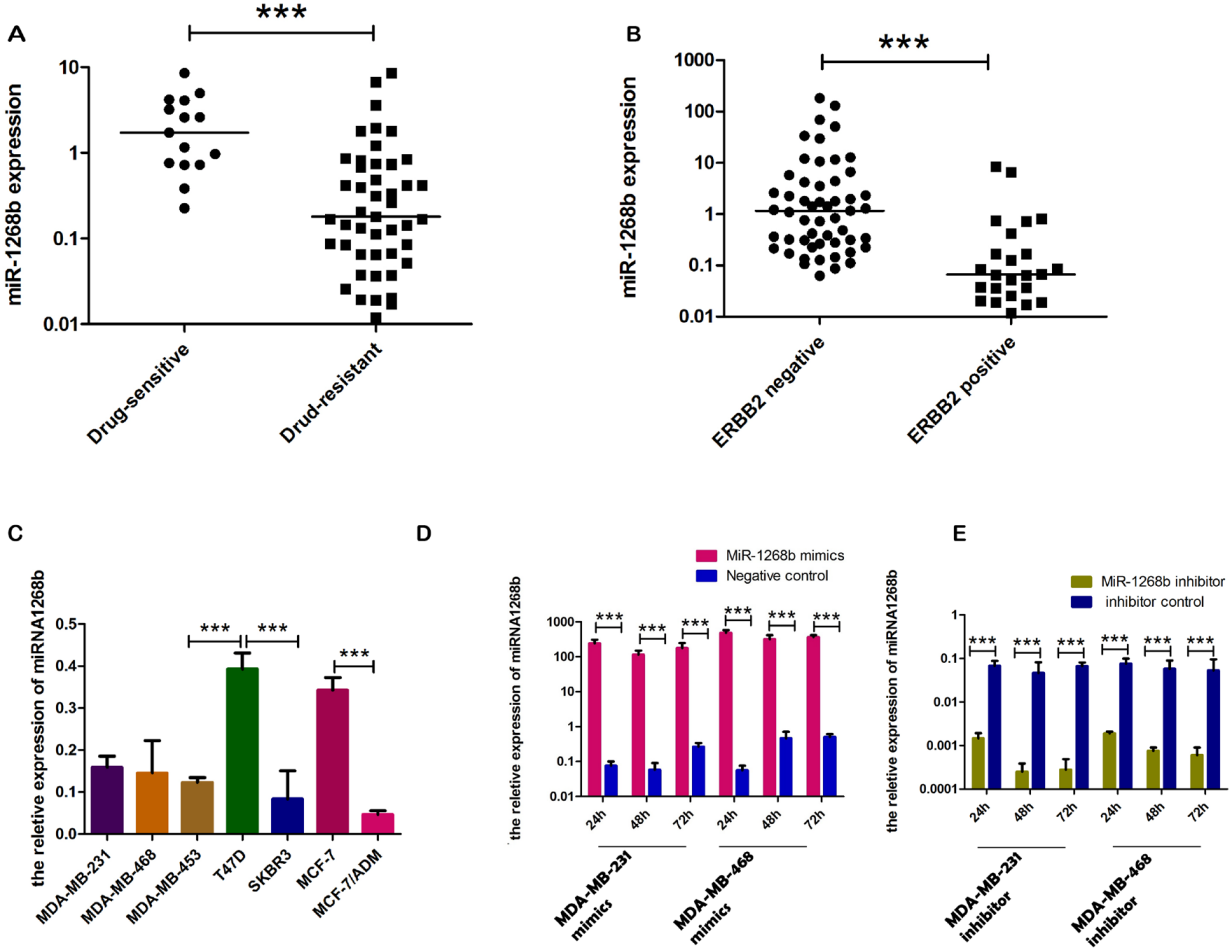
Aberrant expression of miR-1268b in tissues and cell lines **(A)** Determined by RT-qPCR, miR-1268b was significantly upregulated in chemosensitive tissues by 9.6–fold compared with that of chemoresistant tissues (^***^p<0.0001). **(B)** miR-1268b was upregulated by 17.5–fold in ERBB2 negative tissues compared with that of ERBB2 positive tissues (^***^p<0.0001). **(C)** The expression of miR-1268b was detected in seven breast cancer cell lines. MiR-1268b was lower expressed in MCF-7/ADM (resistant to adrimycin), MDA-MB-453 and SKBR3 (Both are ERBB2 overexpression cells) in comparision with other cancer cells. In fact, the expression of miR-1268b in MCF-7/ADM was lower by 7.9-fold than that of MCF-7 cells (^***^p < 0.0001). **(D, E)** After transfected with miR-1268b mimics, expression of miR-1268b was significantly upregulated both in MDA-MB-231 and MDA-MB-468 cell lines, and transfected with miR-1268b inhibitor showed the opposite effect (^***^p < 0.0001).

The expression level of miR-1268b was determined byRT-qPCR in 95 cases of breast cancer tissues. The results revealed that miR-1268b was associated significantly with cancer chemosensitivity and ERBB2 expression, but not with lymph node metastasis or tumor size (Table 1). In fact, miR-1268b was significantly upregulated by 9.6-fold in drug sensitive group compared to the chemoresistance group (Figure 1A), and was upregulated by 17.5-fold in ERBB2 negative group compared to the positive group (Figure 1B). And miR-1268b was highly expressed in patients with lower TNM stage compared with those with advanced TNM stage (Table 1). The endogenous expression of miR-1268b was upregulated in chemosensitive and ERBB2 negative cell lines compared with chemoresistant and ERBB2 positive cell lines, and especially upregulated by 7.9-fold in MCF-7 compared with the drug resistant cell line MCF-7/ADM (Figure 1C). All the data demonstrated that miR-1268b may act as a negative regulator of chemoresistance and ERBB2 expression.

**Table 1:** Correction between miR-1268b and clinicopathological features

Variables	n(95)	miR-1268b expression		P value
		Low	High	
**Age(years)**				
<50	45	21	24	0.2338
≧50	50	24	26	
**Tumour size(cm)**				
<3	51	25	26	0.6207
≧3	44	20	24	
**Lymph node metastasis (LNM)**				
Negative	39	19	20	0.1751
Positive	56	28	28	
**Chemosensitivity**				
Drug-resistant	15	7	8	0.0021
Drug sensitive	23	19	4	
**pT staged**				
T1	23	10	13	0.5193
T2	60	31	29	
T3	4	2	2	
T4	8	4	4	
**Dustant metastases**				
M0	87	43	44	0.5111
M1	8	3	5	
**pTNM stage**				
I	18	9	9	0.0339
II	38	18	20	
III	27	10	17	
IV	12	6	6	
**ER status**				
Negative	32	17	15	0.4473
Positive	63	31	32	
**PR status**				
Negative	34	17	17	0.8431
Positive	61	30	31	
**HER2 status**				
Negative	53	31	22	< 0.0001
Positive	24	11	13	
**ki-67**				
Negative	24	12	12	0.9556
Positive	71	35	36	

### MiR-1268b had no effect on cell proliferation activity of breast cancer

**Figure 2:**
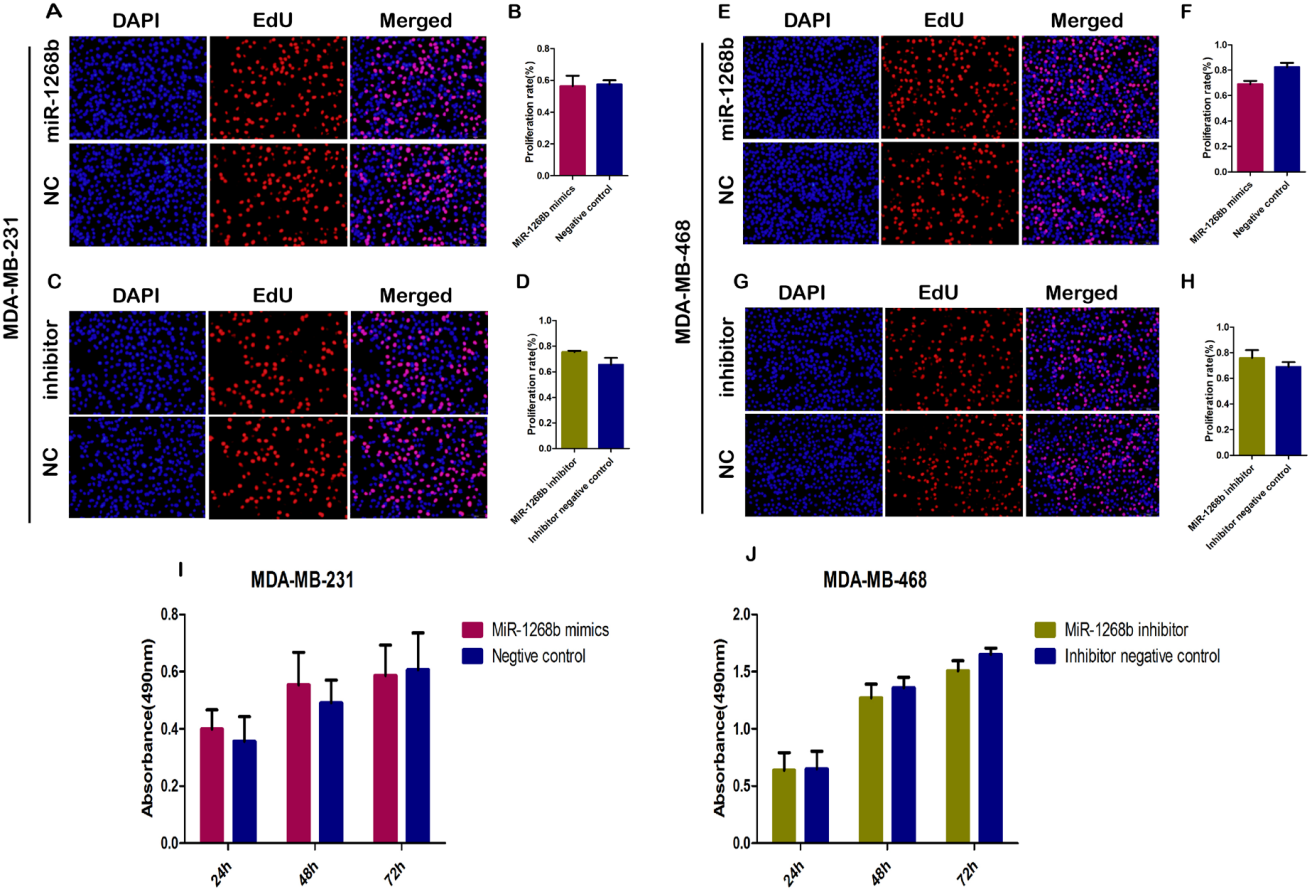
MiR-1268b had no effect on the proliferation ability of breast cancer cells **(A, B, E, F)** EDU assay showed that overexpression of miR-1268b had no effect on the proliferation ability of breast cancer both in MDA-MB-231 and MDA-MB-468 cells. **(C, D, G, H)** It showed that cell number of MDA-MB-231 and MDA-MB-468 in proliferative phase had no significant change when downregulated the expression of miR-1268b in EDU assay. **(I, J)** CCK8 assays further confirmed that miR-1268b had no effect on the cell growth of breast cancer.

To detect the transfection efficiency, RT-qPCR was used to determine the expression of miR-1268b at 24h, 48h, 72h after transfection. Results revealed that transfection with miR-1268b mimics or inhibitor could efficiently induced overexpression or downregulation of miR-1268b compared with the negative control group in both MDA-MB-231 and MDA-MB-468 cell lines (Figure 1D, 1E). To investigate the role of miR-1268b in cell proliferation, CCK8 and EDU assay were conducted. No significant difference was observed either between the overexpression group and the control group or between the downexpression group and the control group (Figure 2A-2J, Supplementary Data A, B).

### MiR-1268b had no effect on cell migration or invasion ability of breast cancer

**Figure 3:**
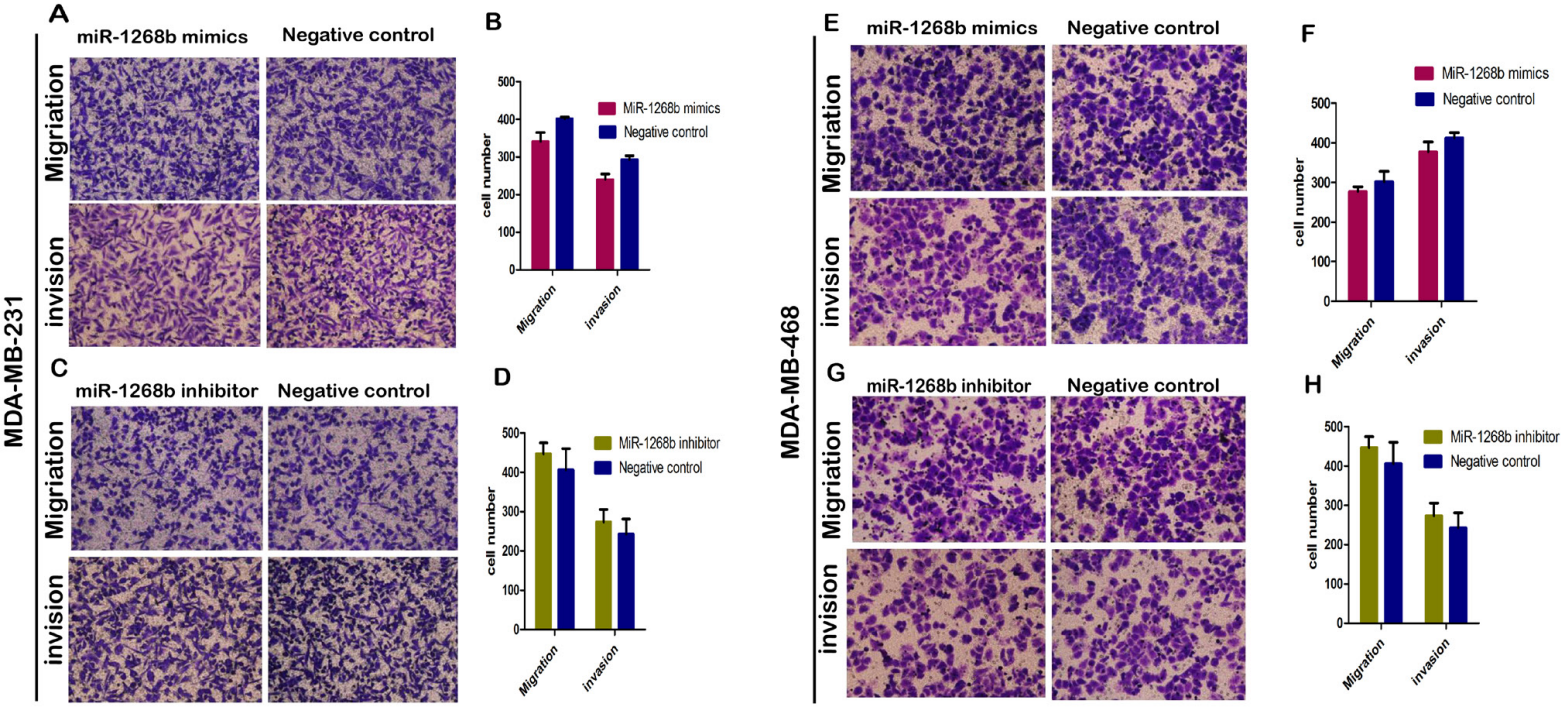
Transwell assay revealed that miR-1268b had no effect on migration or invasion ability of breast cancer **(A-D)** After overexpression of miR-1268b both in MDA-MB-231 and MDA-MB-468 cells, no significant difference was detected both in migration and invasion assays. **(E-H)** The same effect was detected in miR-1268b down regulated cells.

Besides the ability of cell proliferation, other malignant phenotypes of breast cancer cells such as the migration and invasion ability were determined. Transwell assays were performed to detect the role of miR-1268b in cell migration and invasion. Results showed that upregulation or downregulation of miR-1268b did not have a meaningful effect on cell's migration or invasion capabilities compared with the negative control groups both in MDA-MB-231 and MDA-MB-468 cell lines (Figure 3A-3H), suggesting that miR-1268b had no effect on cell migration or invasion of breast cancer.

### MiR-1268b induced apoptosis in breast cancer

**Figure 4:**
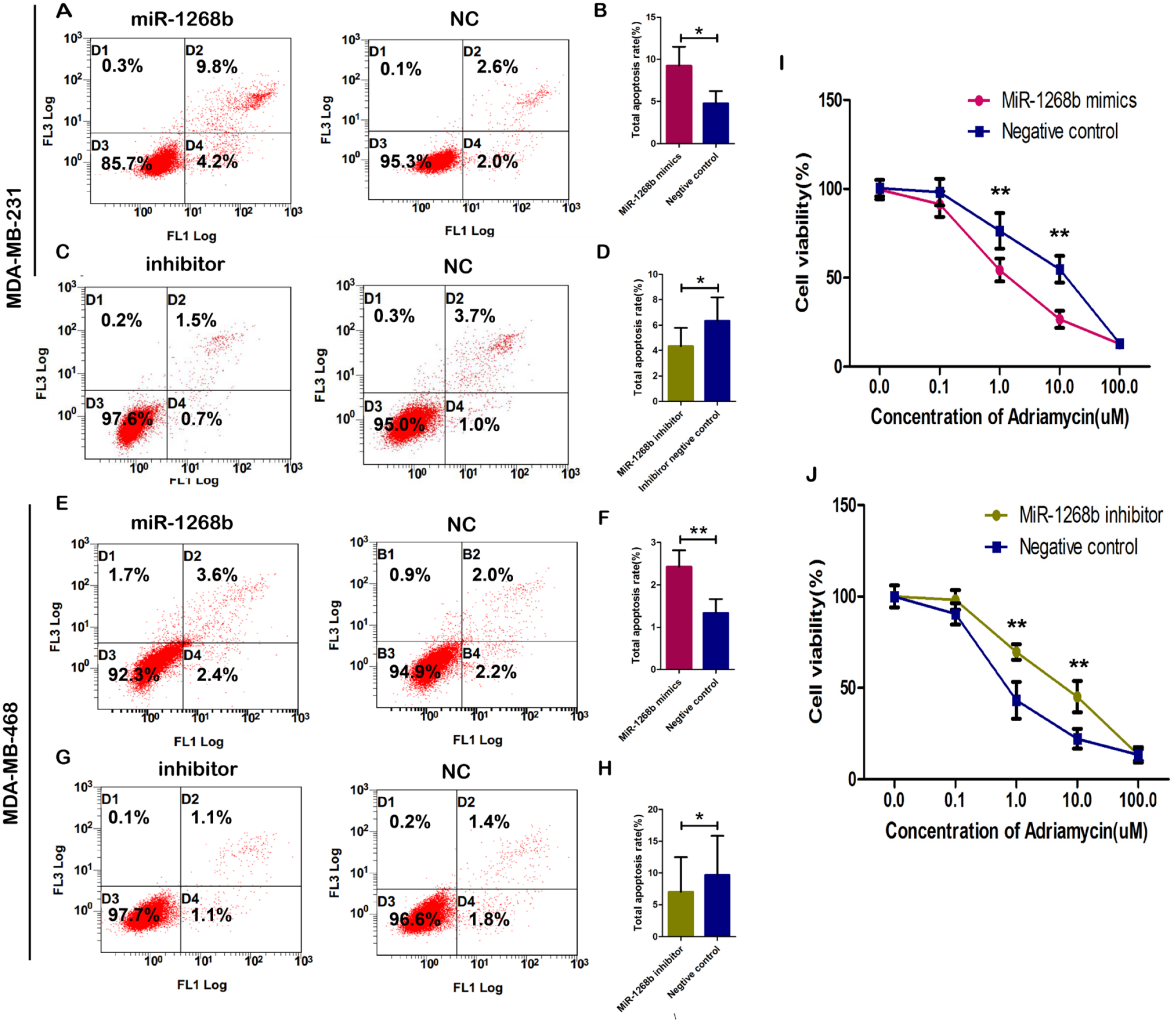
MiR-1268b induced apoptosis and enhanced chemosensitivity in breast cancer **(A, B, E, F)** MDA-MB-231 and MDA-MB-468 cells were labeled with Annexin V-FITC/PI and detected by flow cytometry. The results showed that miR-1268b overexpression could significantly promote the cell apoptosis (^*^p <0.05, ^**^p <0.01). **(C, D, G, H)** MiR-1268b downregulation inhibited the cell apoptosis (^*^p <0.05). **(I)** MCF-7/ADM cells were treated with stepwise concentrations of adriamycin, forty eight hours after transfection. CCK8 assay was performed to detect cell viability and the results showed that overexpression of miR-1268b could reduce cell viability. **(J)** Downregulation of miR-1268b enhanced cell viability.

Flow cytometry (FCM) assay was performed to further detect the effect of miR-1268b on apoptosis of breast cancer cells. The results demonstrated that the group transfected with miR-1268b mimics had a significantly higher proportion of apoptotic cells than that of the negative control group in both MDA-MB-231 and MDA-MB-468 cells (Figure 4A, 4B, 4E, 4F). While miR-1268b inhibitor reduced the apoptosis rate of the transfected cells (Figure 4C, 4D, 4G, 4H). Taken together, these data indicated that miR-1268b could induce apoptosis of breast cancer cells.

### MiR-1268b increased chemosensitivity in breast cancer cells MCF-7/ADM

As the relative expression of miR-1268b is significantly higher in drug sensitive tissues than that of drug resistant tissues, we wonder whether it could induce chemosensitivity in breast cancer cells. MCF-7/ADM cells transfected with miR-1268b mimics/inhibitor or their negative control were added with stepwise increased concentrations of adrinamyin and detected by CCK8 assay. The results revealed that high expression of miR-1268b conferred adrinamyin sensitivity to breast cancer cells compared with the negative control group (Figure 4I), while down regulation of miR-1268b increased the resistance of cancer cells to adrinamyin (Figure 4J). In addition, overexpression of miR-1268b could decrease IC50 by 5.3-fold. While downregulation of miR-1268b increased IC50 by 5.4-fold. All the data indicated that miR-1268b could increase breast caner's sensitivity to adrinamyin.

### ERBB2 was a direct target gene of miR-1268b

**Figure 5:**
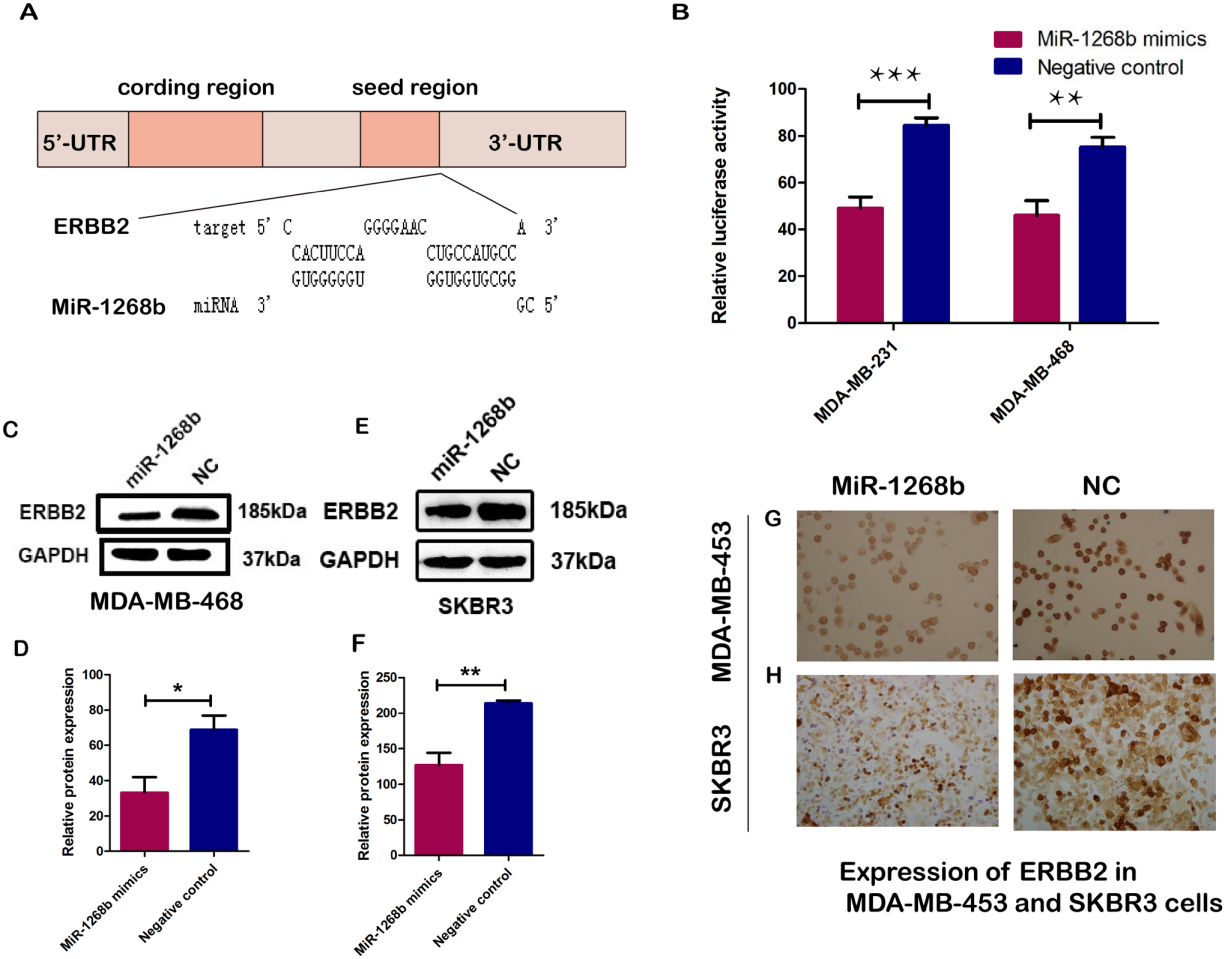
ERBB2 is a direct target of miR-1268b **(A)** The software RNAhybrid predicted that ERBB2 was a potential target gene of miR-1268b, with the binding sites displayed. **(B)** The luciferase activity was reduced in pmirGLO-ERBB2 and miR-1268b co-transfected breast cancer cells in both MDA-MB 231 and MDA-MB-468, in comparison with that of the negative control (^**^p < 0.01, ^***^p < 0.001). **(C, D, E, F)** Western blot assay revealed that overexpression of miR-1268b could dramatically reduce the protein level of ERBB2 in MDA-MB-453 (C, D) and SKBR3 (E, F) (^*^p < 0.05, ^**^p < 0.01). **(G, H)** The result of immunocytochemistry assay showed that miR-1268b transfection, rather than the negative control, could repress the protein expression of ERBB2 in MDA-MB-453 and SKBR3 cells.

Amplication of the ERBB2 gene or overexpression of ERBB2 protein was found in 30% of human breast carcinoma, and proved to result in poorer prognosis [10]. It was also verified that ERBB2 was associated with apoptosis and chemoresistance of breast cancer [11–15]. So ERBB2 protein expression was determined by immunohistochemistry and the cases were divided into ERBB2 positive group and ERBB2 negative group. Then the association between ERBB2 expression and miR-1268b expression was analyzed. The results showed that ERBB2 positive group had significantly lower miR-1268b expression compared with ERBB2 negative group. Additionally, bioinformatics databases miRWalk (http://zmf.umm.uni-heidelberg.de/apps/zmf/mirwalk/index.html) and RNAhybrid (http://bibiserv.techfak.uni-bielefeld.de/rnahybrid/) analysis showed that there were several evolutionary conserved binding sites of miR-1268b in the 3’UTR of ERBB2 (Figure 5A). To further investigate whether ERBB2 is a target gene of miR-1268b, the PmirGLO-ERBB2 was constructed and transfected into two breast cancer cell lines (MDA-MB-231 and MDA-MB-468) together with miR-1268b mimics or negative control. Then, luciferase report assay was utilized. As showed in the results, a significant decrease of luciferase viability was observed in the miR-1268b transfected cells. The viability of luciferase in miR-1268b groups decreased about 36% or 32%, respectively, in MDA-MB-231 or MDA-MB-468 compared with that of the negative control groups (Figure 5B). To further determine whether miR-1268b expression could suppress the protein expression of ERBB2, western blot and immunocytochemistry assays were performed in ERBB2 overexpression cells (MDA-MB-453 and SKBR3). The results showed that miR-1268b could significantly reduce the protein expression of ERBB2 (Figure 5C, 5D, 5E, 5F, 5G, 5H). Taken together, all the data demonstrated that ERBB2 is a direct target gene of miR-1268b.

### ERBB2, PI3KCA, AKT, Bcl2 in the ERBB2-mediated PI3K-AKT signaling pathway were downstream effectors of miR-1268b

**Figure 6:**
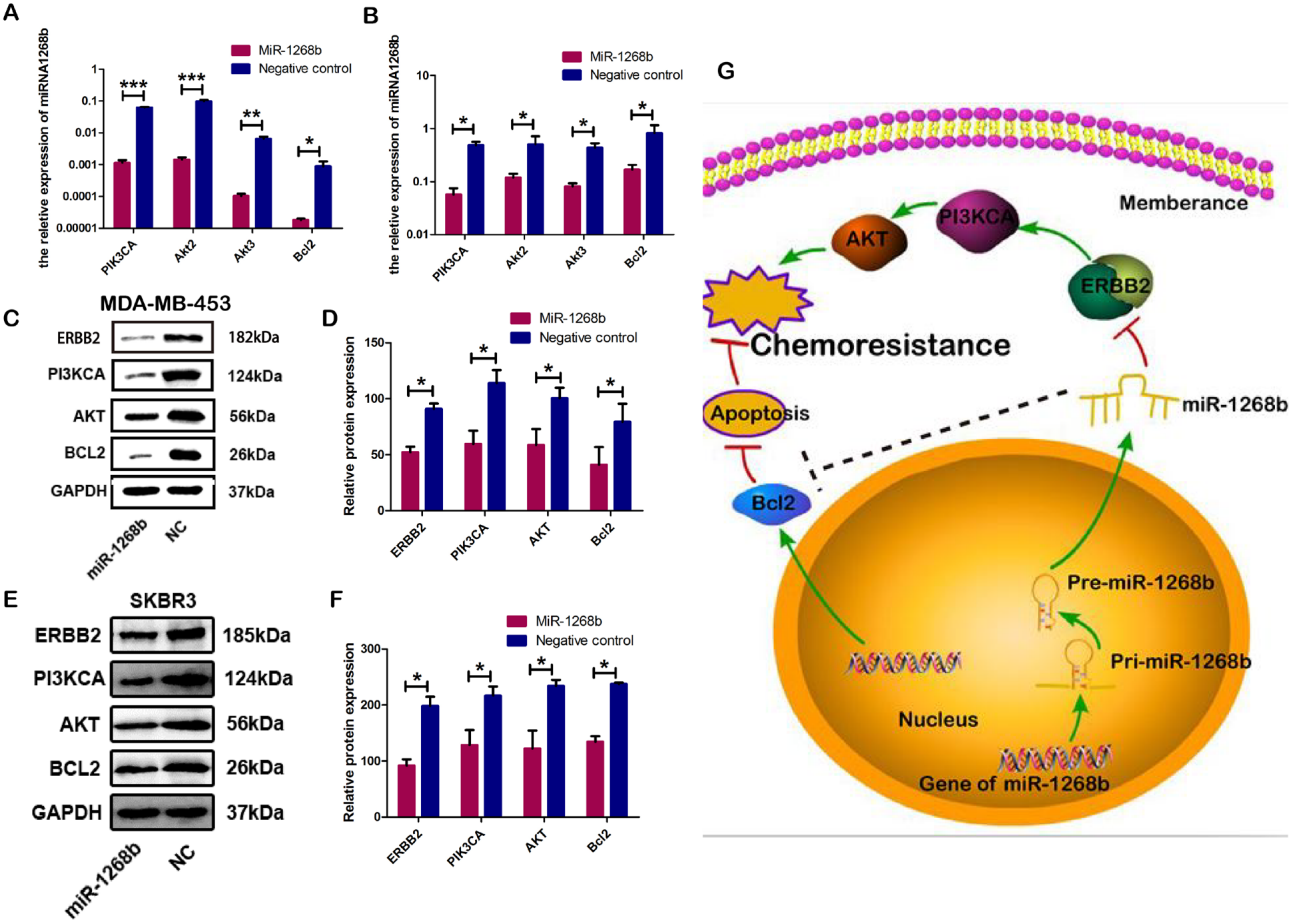
MiR-1268b could suppress the ERBB2-mediated PI3K-AKT signaling pathway **(A, B)** RT-qPCR results showed that miR-1268b could inhibit the expression of PIK3CA, AKT2, AKT3 and Bcl2 in MDA-MB-453 (A) and SKBR3 (B) (^*^p <0.05, ^**^p <0.01, ^***^ p<0.001). **(C, D, E, F)** Western blot assay further showed that miRNA-1268b reduced the protein expression of PIK3CA, AKT and Bcl2 in MDA-MB-453 (C, D) and SKBR3 (E, F) (^*^p < 0.05). **(G)** The schematic diagram showed the potential functional mechanism of miR-1268b to induce chemosensitivity in breast cancer. MiR-1268b could repress the PI3K-AKT signaling pathway by targeting ERBB2 and inhibit the anti-apoptosis protein Bcl2.

PI3K-Akt pathway was reported to be one of the main downstream messengers of ERBB2 signaling [16]. To elucidate whether miR-1268b could regulate the PI3K-AKT pathway, the mRNA and protein level of PI3K/AKT pathway were determined by RT-qPCR and western blot both in MDA-MB-453 and SKBR3. The results showed that overexpression of miR-1268b could suppress the mRNA expression of PI3KCA, AKT2/3, Bcl2 (Figure 6A, 6B). Furthermore, we proved that miR-1268b could decrease the protein expression of ERBB2, PI3KCA, AKT, Bcl2 (Figure 6C, 6D, 6E, 6F). As ERBB2 is a direct target gene of miR-1268b, all the data demonstrated that PI3K/AKT signaling were downstream effectors of miR-1268b, which, at least in part, was induced by targeting ERBB2.

## DISCUSSION

Chemoresistance remains the critical cause of treatment failure and tumor progression for patients with cancers [17]. Despite recent advances, the mechanisms of drug resistance involved in breast cancer are still unclear. It was reported that chemotherapeutic agent against breast cancer, such as adriamycin and paelitaxel, kills tumor cells by inducing DNA, RNA damage and apoptosis [18]. Finding the underlying mechanisms of drug resistance may provide an effective therapeutic method against chemoresistance.

The crucial roles in tumors make miRNAs potential novel anti-neoplasm therapeutic tools like siRNAs [19]. Admittedly, based on the important roles of miRNAs in regulation of various cancers, we have the reason to believe that miRNAs could become potential biomarkers for early tumor diagnosis, prognosis assessment and the modification of anticancer treatments [20–22]. Notably, more and more researches revealed that miRNAs were involved in the chemoresistance of cancer cells [23–27]. These reports suggested that understanding the mechanism of miRNAs involved in chemoresistance of cancers is necessary and promising. Chen et al [28] demonstrated that promotion of SPIN1-mediated PI3K-AKT pathway by miR-489 enhanced chemoresistance in breast cancer. Wang et al [29] showed that miR-497 could serves as a tumor suppressor by suppressing KSR-mediated MAPK/ERK singling pathway. These data indicated the roles and mechanisms of miRNAs modulating chemoresitance in cancers.

MiR-1268b is a new miRNA that has never been investigated in human cancers. In the present study, microarray and RT-qPCR analysis revealed that miR-1268b expression level was upregulated in drug sensitive breast cancer tissues compared with that of drug resistant tissues. Then, we showed that miR-1268b overexpression promoted cells apoptosis and sensitized chemosensitivity to adriamycin in breast cancer. The main mechanism of chemotherapeutic drugs exerting their anticancer effects is to induce apoptosis [30]. To further verify the relationship of miR-1268b and chemosensitivity of breast cancer, we performed cell drug sensitivity experiments, and the results demonstrated that miR-1268b could increase the sensitivity of MCF-7/ADM to adriamycin, as well as the apoptosis of breast cancer cells. All the data showed that there was an interesting relationship between miR-1268b and chemosensitivity of breast cancer.

Next, we analyzed the association between miR-1268b expression level and patients’ clinicopathological parameters. The results showed that miR-1268b was significantly upregulated in ERBB2 negative groups. To investigate the molecular mechanism of miR-1268b in enhancing chemosensitivity of breast cancer, we explored the potential target gene of miR-1268b by softwares of miRWalk and RNAhybrid, and ERBB2 was selected for further confirmation. Dual-luciferase reporter assay, western-blot and immunocytochemistry demonstrated that ERBB2 was a direct target gene of miR-1268b. ERBB2, which is also named as HER2, NEU, belongs to the epidermal growth factor receptor (EGFR) family. Gene amplication, over-expression or transcriptional deregulation of this oncogene is strongly associated with shorter recurrence time and worse prognosis in breast cancers [31], as well as in other cancers like carcinomas of stomach [32], ovarian [33], lung [34]. It also has been verified that ERBB2 is involved in apoptosis of breast cancer cells [35]. ERBB2 encodes a growth factor receptor (185-kDa transmembrane glycoproteins) on the cell surface whose activation drives downstream signaling pathway like Ras-MARK, PI3K-AKT [36]. Nowdays, new treatments like anti-ERBB2 targeted therapies have radically improved the patients’ survival for ERBB2 positive breast cancers [37].

To further elucidate the underlying mechanisms of miR-1268b-mediated drug sensitivity. Downstream effectors of ERBB2 including PI3K/AKT signaling pathway cascades were identified. The ERBB2-PI3K-AKT axis has been well established in breast cancer. Aberrant activation of ERBB2 and PI3K-AKT pathway is associated with tumorigenesis, carcinoma progression and drug resistance [38, 39]. Some biomarkers like microRNAs targeting PI3K-AKT pathway are expected in either preclinical or clinical stages for therapy of tumors like breast cancer. Our results showed that the forced expression of miR-1268b could significantly reduce the expression of PI3KCA, AKT, Bcl2 in PI3K-AKT pathway both in mRNA and protein levels. And interestingly, consistent with the function of miR-1268b to induce apoptosis, the anti-apoptosis protein Bcl2 was significantly down-regulated by miR-1268b. Hence, our results suggested that overexpression of miR-1268b confers chemosensitivity in breast cancer by suppressing ERBB2-mediated PI3K-AKT pathway and inducing apoptosis (Figure 6D).

In conclusion, our results showed, for the first time, that miR-1268b could reverse chemoresistance of breast cancer, at least partly, by targeting ERBB2 and suppressing PI3K/AKT pathway. Clarifying in-depth the mechanisms of drug resistance help to find new effective treatments for reversal of chemoresistance. MiRNA-1268b / ERBB2 / PI3K signaling may become a potential target for reversing the chemoresistance of breast cancer.

## MATERIALS AND METHODS

### Human breast cancer specimens

The formalin-fixed paraffin-embedded (FFPE) breast cancer specimens were obtained from Qilu Hospital of Shandong University (Jinan, China) during 2008 to 2013. The clinical data were collected from patients whose diagnoses were confirmed by two independent experienced pathologists. Drug sensitive (DS) and drug resistant (DR) groups were established according to the Miller-payne grade system (grades 1, 2 were considered to be DR and grades 3, 4, 5 were considered to be DS) [40]. And this research was approved by Research and Ethics committee at school of medicine, Shandong University, China.

### Cell culture and reagents

Human breast cancer cell lines MDA-MB-231, MDA-MB-468, MDA-MB-453 SKBR3 (MDA-MB-453 and SKBR3 are ERBB2 positive cell lines), MCF-7 were acquired from the American Type Culture Collection (ATCC) and maintained in Leibovitz's L15 medium supplemented with 10% fetal bovine serum (FBS, BI, Grand Island, NY, USA.). MCF-7/ADM cells were derived by treating MCF-7 cells with stepwise increasing concentrations of Adriamycin and maintained in the presence of a low concentration of Adriamycin and passaged for one week in drug-free medium before the experiments. All cell lines were incubated in a 5% CO2 humidified atmosphere at 37°C. Drug resistant cell line ADM was cultured with continuous treatment of adrimycin (Dalian Meilun, China) to maintain the chemoresistance until one week before the experiments.

Cell lines were transfected with miR-1268b mimics/ negative control (NC), or miR-1268b inhibitor/inhibitor negative control by X-tremeGENE transfection reagent (Roche, USA). Plasmid (assembled with 3’UTR of ERBB2) was transfected to cells using tuberfect tansfection regent (Roche, USA).

### RNA extraction and RT-qPCR assay

After cut into 4-μm slices, dewaxed, rehydrated and stained with hematoxylin, FFPE tissues were disposed for microRNAs extraction using a miRNA rapid extraction kit (Bioteke, Beijing, China). For cell lines, total RNA was isolated from harvested cells using Trizol reagent (Invitrogen, USA). Reverse transcription PCR and quantitative PCR (RT-qPCR) kit (GeneCopoeia of Shanghai, China) were used to detect the relative amount of RNA according to the manufacturer's instructions, using RNU6B as endogenous control.

### Proliferation assay

The Proliferation assays were monitored using cell-counting kit8 (CCK-8, BestBio, Shanghai, China) and Cell-Light™ EDU DNA Cell Proliferation (EdU: 5’Ethynyl-2’-deoxyuridine) kit (Ribobio, Guangzhou, China) according to the manufacturer's instructions. Cells were seeded in 96-well plates at 5000 per well (CCK-8) or 20000 per well (EDU) 12 hours (in the logarithmic phase of growth) after transfection and incubated at 37°C in a humidified 5% CO_2_ atmosphere for 24h, 48h, 72h (CCK-8) or 12h (EDU) respectively. As for CCK-8 assays, the spectrometric absorbance was read at 490nm using a microplate reader (BIO-RAD, CA, USA). For EDU experiments, a fluorescence microscope (ThermoFisher, USA) was used to count the cells in proliferating phase (excitating bright red spots) and the total cells (blue ones), the proliferation index (PI) was calculated (PI = red spots / blue ones).

### Migration and invasion assays

Transwell chambers with polycarbonate membrane inserts (Corning, NY, USA) were used to conduct cell migration assay. Cells (6×10^^^4 per well) were suspended in 180 μl of serum-free RPMI 1640 or Leibovitz's L15, with 600ul of the same medium (contained 15% FBS) placed in the lower chambers to stimulate cell migration. Sixteen hour’ incubation later, cells adhering to the transwells were fixed with 3% paraformaldehyde for 30 minutes, stained with 0.1% crystal violet for 15 minutes. Cells on the upper chambers were wiped with cotton ball, leaving migration cells sticking to the bottom of chambers. Five random fields were picked at 200× by the camera of inverted microscope (Thermo Fisher Scientific).

For the invasion assays, the transwells were placed with matrigel matrix (BD science, sparks, USA) on the bottom and were put in incubator for 24 h, the other steps were same as migration assays above. All the experiments were repeated three times.

### Apoptosis and flow cytometry analysis

Cell apoptosis was performed using an Annexin V-FITC apoptosis detection kit (BestBio, Shanghai, China) following the manufacturer's protocols. Briefly, the cells were seeded in 6-well plates at a density of 1.5×10^5 per well, forty eight hours after transfection, cells were digested by 0.08% EDTA-free trypsin and washed with ice-cold PBS twice. Application of porpodium iodide (PI) and Annexin V-fluresceinisot hiocyanate (FITC) staining determined the percentage of cells undergoing apoptosis or necrocytosis. Cell apoptosis were measured using the flow cytometry (Beckman coulter, KBB, CA, USA) and data were analyzed by FlowJo software.

### Dual-luciferase reporter assay

The ERBB2 3’-UTR fragment containing the predicting miR-1268b binding sites were amplified by PCR using PrimerSTAR Max DNA polymerase (Takara, Japan) and primers: 5’ GCGGA GCTCAACCAGAAGGCCAAGTC 3’ (FORWARD), 5’ GCGTCTAGAAAATGGACAAAGTGGGTGTGG 3’ (REVERSE) designed and synthesized by company of biosune (Jinan, China). The 3’-UTR of ERBB2 were then inserted into the XbaI and SacI sites of pmirGLO target expression vector (Promega, San Luis Obispo, CA, USA.).

Breast cancer cells MDA-MB-231 and MDA-MB-468 (at a density of 5×10^4 per well) were seeded into 24-well plates 12h before transfection. Then, the cells transfected with 1ug of ERBB2 3’UTR fragment reporter vector containing the predicted miR-1268b binging sites, co-transfected with 30nM miR-1268b mimics or negative control using Tuberfect tansfection reagent (Roche, USA). After 48h’ incubation, cells were lysed by PLB (1×,100ul) and were put on an orbital shaker (QILINBEIER, Jiangsu, China) at 100rpm for 30 minutes before placed in -80°C refrigerator overnight. renilla luciferase (internal reference) and firefly luciferase signals were measured using a dual-luciferase reporter assay kit (Promega, Madison, WI, USA.) according to the manufacture’ s instructions.

### Western-blot assay

Fifty μg of protein was electrophoresed in 10% sodium dodecyl sulfate polyacrylamide gel electrophoresis (SDS-PAGE) and transferred to a polyvinylidene difluoride membrane (PVDF, EMDMillipore, MA, USA), which was probed with antibodies to ERBB2. The blots were detected and visualized by an enhanced chemiluminescence detection kit (Millipore, Billerica, MA, USA) according to the manufacture's protocols.

### Immunohistochemistry

Paraffin-embedded blocks were cut into 4 μm thick sections and subjected to deparaffinized re-hydrated, and immersed in double distilled water with hydrogen peroxidise (3%) to reduce endogenous oxidise activity. As for cell immunohistochemistry, cells were seeded in 6-well plates with coverslips pre-coated at the bottom, forty eight hours after transfection, coverslips adhered with cells were fixed with 4% paraformaldehyde for 30 minutes and washed with PBS for three times. Either tissue or cell immunohistochemistry were then incubated with primary antibody at 4°C for 1h, followed by secondary antibody applied to cells at room temperature for 30 minutes. Staining degree was developed by diaminobenzidine (DAB) chromogen (Dako, Inc, carpinteria, CA, US) and cell nucleuses were dyed with hematoxylin for 30s. Subsequently, the tissues or coverslips were dehydrated and sealed with gum. According to the modified Guideline Recommendations for Human Epidermal Growth Factor Receptor 2 (HER2) Testing in Breast Cancer [41, 42], the immunoreactivity was graded by scoring the percentage of positive membrane staining: negative (0, 1+), equivocal (2+), positive (3+), as previously reported.

### Chemosensitivity assay *in vitro*

Cells were seeded in 96-well plates at a density of 5×10^3^ per well and were cultured for 16 hours after transfection with either miR-1268b/NC or miR-1268b inhibitor/inhibitor control, and then treated with increasing concentration of adrinamyin ranging from 0 to 100μM, 5 replicate wells for each concentration. Twenty four hours later, cell absorbance was assayed at 490nm using CCK8 kit. IC50 (Half maximal inhibitory concentration), which was defined as the concentration at which cell growth was reduced to 50% according to the relative survival curve was calculated.

### Statistical analysis

GRAPH PAD prism software 5.0 (Graphpad, software, Inc, LaJolla, CA, USA) were utilized for statistical analyses. Comparasion between two groups was subject to t-test, while those multiple groups with analysis of variance (ANOVA). All experiments were performed at least 3 replicates and data were presented as the mean±standard deviation (S.D). A p-value < 0.05 was statistically significant.

## SUPPLEMENTARY MATERIALS FIGURES AND TABLES


