# Supplementary material for: MiR-1268b confers chemosensitivity in breast cancer by targeting ERBB2-mediated PI3K-AKT pathway
Source: Oncotarget. 2017 Aug 9;8(52):89631–42. doi: 10.18632/oncotarget.20099 (PMC5685697; doi:10.18632/oncotarget.20099)
Supplement: Supplementary file 1 [file oncotarget-08-89631-s001.pdf]

MiR-1268b confers chemosensitivity in breast cancer by targeting ERBB2-mediated PI3K-AKT pathway

SUPPLEMENTARY MATERIALS

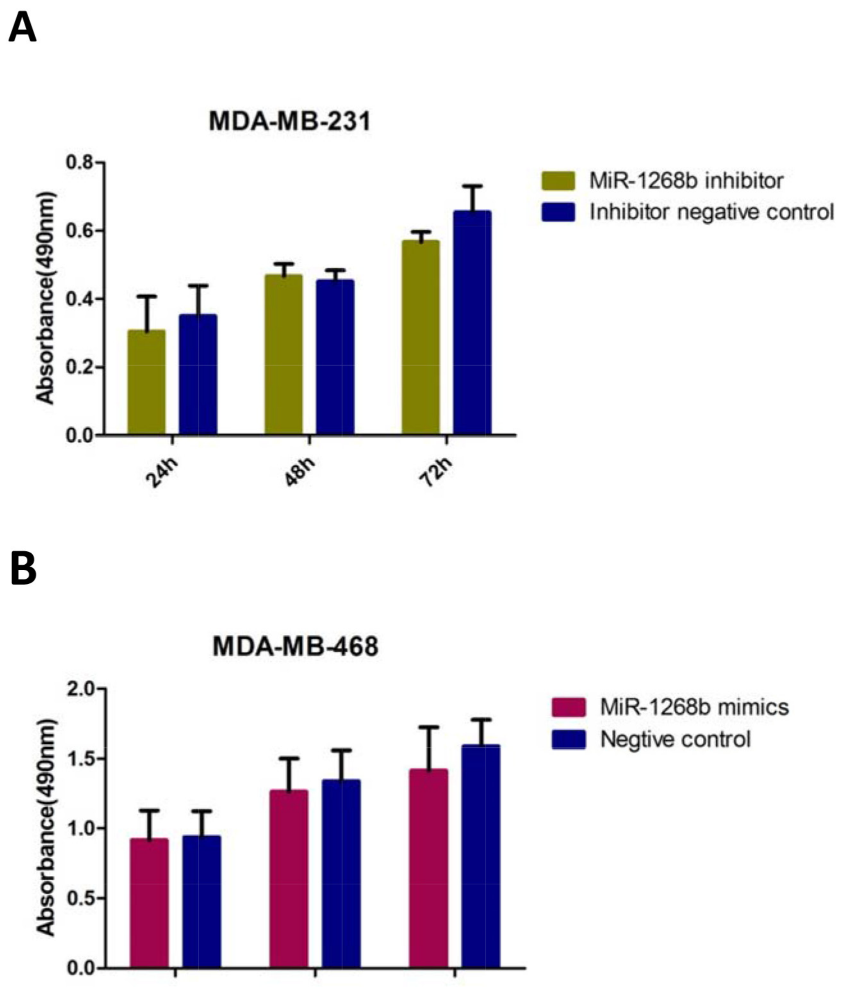

Supplementary Data: CCK8 assay in breast cancer cells. **(A, B)** Inhibit or uprugulate the expression of miR-1268b have no effect on cell proliferation ability in MDA-MB-231 and MDA/MB-468 cells.
